# Hurdles to boys with acute scrotal pain being evaluated and treated in district general hospitals: we are not Getting It Right the First Time yet

**DOI:** 10.1308/rcsann.2024.0078

**Published:** 2025-04-03

**Authors:** M Gopal, U Ibrahim, I Salphale, M Mostafizur, S Walker, M Clement, D Macafee, A Patel, R Balu, KS Eswaravaka Sudha Radha

**Affiliations:** ^1^ The Newcastle upon Tyne Hospitals NHS Foundation Trust, UK; ^2^ County Durham and Darlington NHS Foundation Trust, UK; ^3^ South Tyneside and Sunderland NHS Foundation Trust, UK; ^4^ South Tees Hospitals NHS Foundation Trust, UK

**Keywords:** Hurdles, Acute, Scrotal, Pain, Evaluation

## Abstract

**Introduction:**

Testicular torsion is a time-critical emergency, though its incidence in the paediatric age group is relatively rare. Changes in training pathways have led to a decreasing number of adult general surgical and urological trainees being comfortable in performing an emergency scrotal exploration in children, resulting in children being transferred to regional units with the requisite expertise. This delay has been shown to increase the risk of orchidectomy. There is, therefore, an increased emphasis on the diagnostic evaluation of these children by emergency department staff.

**Methods:**

We explore how lack of experience and availability of appropriate investigations led to transfer of children presenting to a district general hospital in the North East of England.

**Results:**

Children with true testicular torsion in this cohort had an unacceptably high rate of needing an orchidectomy (∼67%) compared with the reported incidence of orchidectomy with testicular torsion (∼20%).

**Conclusions:**

We offer potential solutions to the hurdles that have to be overcome to improve this service. This will align the service to the recently published Get It Right First Time report on the management of testicular torsion in children and young adults and is within the remit of Operational Delivery Networks.

## Introduction

Testicular torsion (TT) is a time-critical emergency. Testicular salvage rates are optimal if operated on within 6–8h of onset of symptoms.^1^ Health education is vital to encourage early presentation to local healthcare services.^2^ Once at the district general hospital, the emergency department (ED) staff have several questions to answer:

• Does this child with acute scrotal pain have a TT?
• Are there local provisions for Doppler ultrasound (DUS) evaluation either by ED staff as point of care ultrasound (POCUS) or by radiology?
• Is there in-house surgical (general surgery or urology) and anaesthesia provision?
• Should these children be transferred to the nearest paediatric surgical centre?

Both Get It Right First Time (GIRFT) and the National Confidential Enquiry into Patient Outcomes and Death (NCEPOD) recently published their recommendations for the management of children and young adults with TT.^3,4^ Some of the key recommendations included:

• avoid/limit transfers
• surgical decision maker to review patient within 60min of arrival at the ED
• use of the Testicular Workup in Suspected Torsion (TWIST) score
• appropriate use of DUS
• children with suspected TT should have surgery within 1h of the decision to operate.

The development of Operational Delivery Networks (ODN) in England was aimed at increasing the delivery of local services.^5^ Using the data of children presenting to the ED at the University Hospitals of North Durham (UHND), we offer a contemporary real-world practice review to assess compliance with national recommendations. We then discuss in a stepwise manner the hurdles to overcome.

## Methods

We retrospectively reviewed the details of all boys aged >6 months and <16 years who presented to UHND (a district general hospital in the North East of England) with acute scrotal pain between September 2019 and August 2021. We recorded the age at presentation, referral source, outcomes and duration of stay in the ED. For boys transferred to the neighbouring surgical centres (paediatric surgery at the Great North Children’s Hospital [GNCH], Newcastle [17 miles away] or adult urology at Sunderland Royal Hospital [SRH; 14 miles away]), we noted the outcome (clinical examination and discharge, clinical examination and surgery, scrotal DUS and discharge, scrotal DUS and surgery), total duration of the episode (from presentation to Durham to discharge or surgery at Newcastle or Sunderland), findings at surgery and whether any patient discharged without surgery represented to the hospital. We then compared these data with the recommendations published by GIRFT on the management of suspected TT in children and young adults.

The use of an online National Health Service (NHS) research tool suggested that this retrospective case note review did not require ethical clearance. This study was registered as an audit (no. 1448) with the local clinical governance body at UHND.

## Results

A total of 53 paediatric patients, aged between 6 months and 16 years (mean age 10.21 years, sd 3.8104), presented to the UHND with testicular pain between September 2019 and August 2021. Referral sources include NHS helpline (36%), general practitioner/practice nurse (13%) and self-referral (46%). The referral source was unknown for two patients (4%). Two of the patients were discharged following assessment by ED staff (one had clinical examination alone and the other had a POCUS scan). Fifty-one patients (96.2%) were referred to specialist centres (10 SRH; 41 GNCH).

**Figure 1:**
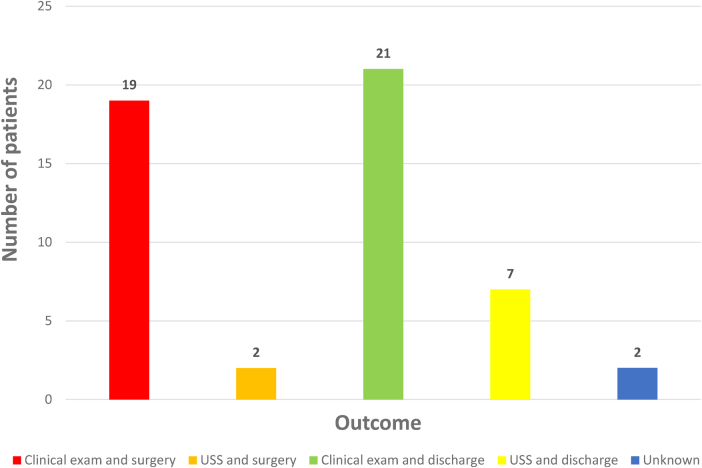
Outcomes of patients transferred to specialist centres

Nineteen (37.2%) patients referred to the specialist centres had surgery following clinical examination, two (3.9%) had DUS then surgery, twenty-one (41.1%) were discharged after clinical examination alone and seven (13.7%) were discharged after DUS (Figure 1). Data for one patient could not be retrieved and another patient self-discharged prior to examination or DUS at the referral hospital.

**Figure 2:**
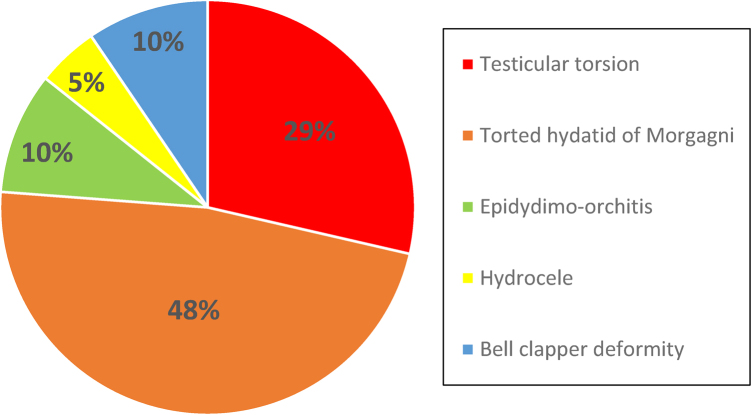
Findings during surgical exploration (per cent rounded to nearest whole number)

Six (28.5%) of patients who underwent surgery were found to have TT, of whom four (67%) required orchidectomy. Other surgical findings included torted hydatid of Morgagni (38%), epididymo-orchitis (9.5%), hydrocele (4.7%) and bell clapper deformity (9.5%) (Figure 2 and Table 1). For patients that were discharged without surgical exploration, none returned with a missed torsion.

**Table 1:** Intraoperative findings at each specialist centre

	Patients transferred to GNCH (*n *= 41)		Patients transferred to SRH (*n *= 10)	
Outcome	Number of patients (%)	Intraoperative findings	Number of patients (%)	Intraoperative findings
Clinical exam and surgery	15 (36.5)	Torted hydatid 7 Testicular torsion 5 Bell clapper deformity 1 Hydrocoele 1 Epididymo-orchitis 1	4 (40)	Torted hydatid 2 Epididymo-orchitis 1 Bell clapper deformity 1
DUS and surgery	2 (4.8)	Testicular torsion 1 Torted hydatid 1	0 (0)	N/A

DUS = Doppler ultrasound; GNCH = Great North Children’s Hospital; N/A = not applicable; SRH = Sunderland Royal Hospital.

**Figure 3:**
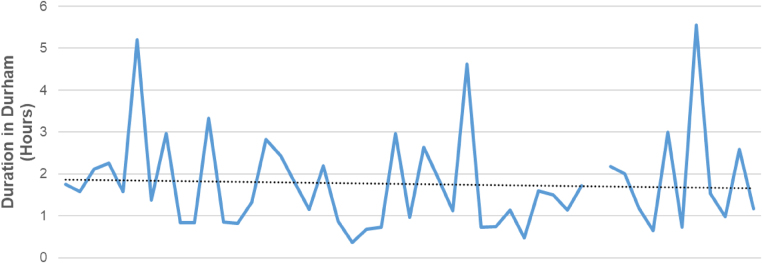
Duration of patient encounter at University Hospitals of North Durham. The dotted line represents the mean duration of 1h 46min.

**Figure 4:**
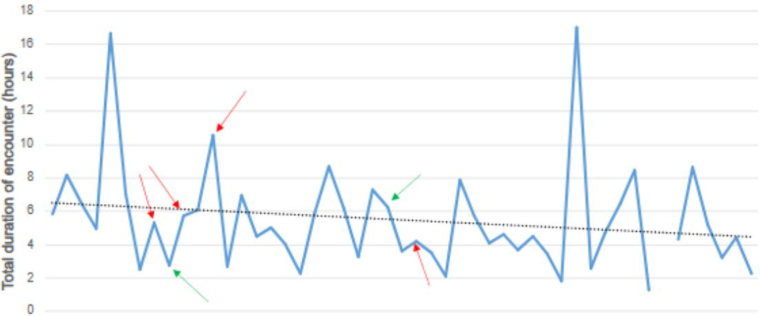
Total duration of encounter from University Hospitals of North Durham to surgery in a specialist centre. The dotted line represents the mean duration of 5h 29min. Arrows indicate the patients with testicular torsion – red arrows led to orchidectomy (mean duration 6h 25min) and green arrows led to testicular salvage (mean duration 4h 53min).

The duration of stay in the ED before transfer to a specialist centre ranged from 22min to 5h 33min (mean 1h 46min; sd 1h 10min) (Figure 3). Among the boys transferred to the regional paediatric surgical centre (GNCH), 41.5% underwent surgical exploration. The average duration from attendance to surgery at GNCH was 3.6h (range 1.7–5.9h). For those referred to SRH, 40% underwent surgical exploration. The average duration from attendance to surgery was 2.5h (range 1.9–3.1h). The total duration of the encounter from arrival at UHND to outcome at specialist centre ranged from 1h 17min to 17h 1min (mean 5h 29min; sd 3h 10min) (Figure 4). The total duration of the encounter for the four patients who required orchidectomy ranged from 4h 13min to 10h 34min (mean 6h 25min), whereas the two patients with TT but viable testes at surgery had duration of encounters of 3h 18min and 6h 29min respectively (mean 4h 53min).

## Discussion

Our review revealed that almost all of the children presenting with acute scrotal pain to the ED of UHND were transferred to regional urology or paediatric surgical centres. This was despite having on-site general surgery and radiology on-call cover. Transfers were done expeditiously, with the mean duration of stay at UHND just under 2h. However, only 41% of the children transferred to the regional centres eventually required surgery. Of these children, only 28.5% revealed TT. Despite a quick transfer, the additional time taken before definitive intervention likely contributed to the high orchidectomy rate of 67%.

ED staff must be cognisant of the fact that the majority of children who present with acute scrotal pain will not have TT. The conundrum is that the transfer of all children to specialist centres is a poor utilisation of resources, and the transfer of those that do have TT may lead to an unacceptably high orchidectomy rate. Our data reveal that there were multiple deviations from the GIRFT recommendations, both at the district general hospital and at the specialist hospitals. With this in mind, let us now try and answer the questions posed at the start of this article.

### Does this child with acute scrotal pain have TT?

TT is a relatively rare surgical emergency with an incidence of around 4 in 100,000 males by 25 years of age.^6,7^ It is seen predominantly in post-pubertal patients with another small peak in the neonatal period. In the UK because of the current reliance only on history and clinical examination and the infrequent use of preoperative ultrasound, only ∼25% of explorations for acute scrotal pain reveal TT. This has been shown both in publications from centres that have a policy of exploring every child with acute scrotal pain and from a recent survey of practice from four paediatric surgical centres in the north of England.^8–10^ History and clinical examination by themselves are therefore poor discriminators of the various causes of acute scrotal pain in children. These include torsion of appendix of testis and epididymo-orchitis, neither of which require emergency surgery.

Validated clinical risk scores have been developed to aid the assessment of acute scrotal pain. The best studied is the TWIST score.^11^

This seven-point score, based on five features from the history and clinical examination, stratifies the risk of TT. When the score is <2, the sensitivity, specificity, positive predictive value and negative predictive value were 100%, 82%, 49% and 100%, respectively. In children in whom the score was >5 these values were 76%, 100%, 100% and 96%, respectively.^11^ The TWIST score has been shown to be useful when performed by ED staff prior to urological consultation and has also been shown to be beneficial in a recent systematic review and meta-analysis.^12,^^13^

Obtaining a TWIST score could, therefore, be a relatively easy tool to adopt and would almost certainly facilitate communication between specialties.

### Are there local provisions for DUS evaluation either by emergency physicians as POCUS or by radiology?

Unfortunately, the role of DUS in the assessment of acute scrotal pain in children in the UK remains controversial. We use the term ‘unfortunately’ because there is a large amount of literature supporting its use and it has become common practice in many countries.^11,14,15^ In 2016 a commissioning guide released by NHS England on the management of paediatric TT stated that “[I]maging studies should NOT be performed as they may delay treatment, therefore prolonging the ischaemic time.^16^ Negative surgical exploration is preferable to a missed diagnosis as all imaging studies have a false-negative rate”. Interestingly, the previous year, the European Uroradiology taskforce released its recommendations of imaging in suspected torsion in which they state “In suspected testicular torsion (after the perinatal period), immediate surgical exploration is advocated when the clinical findings are highly suspicious.^17^ In equivocal cases, immediate ultrasound should be performed without diagnostic delay by an experienced ultrasonographer”*.* Because we know that TT only accounts for ∼25% of children presenting with acute scrotal pain and that UK practice currently has a high negative exploration rate, the role of DUS cannot continue to be ignored. Specific DUS features including the appearance of a twisted spermatic cord (whirlpool sign) have reported >95% sensitivity and specificity in meta-analysis.^15^ Based on the use of TWIST score and DUS, Gopal *et al* devised a clinical algorithm that divides patients into high- and low-risk categories.^10^ After adopting these changes, Gopal *et al* subsequently reported that the percentage of children they were exploring had reduced to 25% and that the incidence of finding TT during emergency exploration had increased from 18% to 53%.^18^ These practice changes show that radiology support with DUS for the evaluation of children who have a moderate clinical suspicion for TT can be achieved in an NHS setting.

POCUS is being increasingly used by ED staff for various conditions. Its use in the assessment of paediatric acute scrotal pain has shown good promise, but as with many operator-dependent diagnostic modalities, its accuracy is based on the training and experience of the user.^19,20^ There is currently a National Institute for Health and Care Research bid in place for funding to evaluate POCUS for acute scrotal pain in the UK. In preparation for this, specific training days on the use of DUS in suspected torsion have been conducted and the results of these have also been published.^21^ Increased training of ED and surgical staff in POCUS for suspected TT would likely aid in its diagnosis.

Of this cohort of boys transferred from UHND to regional centres for suspected TT, 59% did not have surgery. Local provision of DUS or POCUS could have helped to reduce this percentage because many low-risk boys could have been safely discharged without transfer. We appreciate that this is a difficult problem to fix because it involves both a change of mindset with regards to the use of DUS for acute scrotal pain in the UK, and provisions for radiology training and staffing. Of the 41% of transferred children who did have surgery, only 30% revealed TT. This too could have been improved by increased use of DUS at the tertiary centres. By highlighting these issues, the authors hope to motivate readers to begin to instigate changes in their centres. GIRFT recommends DUS only in those children with a strong suspicion of an alternate diagnosis (that maybe suggested by a low TWIST score) and only if it can be done expeditiously. A child should not be transferred to another centre to obtain a DUS.^3^

### Is there in-house surgical (general surgery or urology) and anaesthesia support?

Adult general surgery and urology curricula stipulate competency for the independent management of TT on completion of training.^22,23^ However, a recent report from Wales noted that general surgical trainees had 50% less exposure to emergency scrotal exploration compared with their urology counterparts.^24^ This same report found that <20% of general surgery or urology trainees had any formal exposure to paediatric surgery during either their core or higher surgical training. This lack of training and exposure is a major reason why many current trainees and junior consultants are uncomfortable in performing a scrotal exploration in children, leading to transfer. This is at direct odds with the GIRFT recommendations and the NHS commissioning guide for the management of paediatric TT, which both state that transfer of a patient with suspected torsion should only happen in exceptional circumstances, implying that there should be adequate local expertise to deal with boys with suspected torsion.^3,16,25^

All the boys from the UHND cohort were transferred because most of the general surgical team was not comfortable in the assessment and management of paediatric acute scrotal pain. How can we overcome this conundrum? We believe that knowledge and use of the TWIST score and increased radiology support is vital. However, there is no substitute for experience. We therefore recommend that a minimum 3-month placement in paediatric surgery should be incorporated into the training curricula of both general surgery and urology. The Nottingham ODN successfully ‘decentralised’ the management of paediatric TT, thereby significantly reducing their orchidectomy rate.^26^ They did this by working with their local adult general surgery and urology colleagues to increase their experience and confidence, including the use of wet lab training models. We hope to replicate this in the North East ODN.

None of this can proceed without the local availability of general anaesthesia provision for children. This varies between centres and is contingent not only on staff training and competency of anaesthetists and anaesthetic nurses/operating department practitioners, but also on organisational factors, such as appropriate ward facilities, nursing staff and other requisite support, for example a named consultant paediatrician.^27,28^ National guidance from the Royal College of Anaesthetists and the Royal College of Surgeons of England, should underpin ODN and individual centre agreements regarding emergent paediatric surgery in non-specialist centres. A lower age limit of ≥3 years is generally accepted by most (non-specialist paediatric) anaesthetists for safe elective paediatric work in healthy children, i.e. those without significant comorbidity. This ‘safe’ age threshold may need to be raised according to an individual anaesthetist’s lack of regular exposure to paediatric cases, which in turn impacts their necessary maintenance of paediatric anaesthetic competency and capability.^28^ However, because true TT is rare in a pre-pubertal child, with improved preoperative surgical assessment, most children needing surgery should be significantly older than this 3-year threshold.

### Should these children be transferred to the nearest paediatric surgical centre?

TT a time-critical event, with most cases having a salvageable testis if the duration is <6h and a minimal chance of salvage for durations >24h.^1^ Ninety-six per cent of children with acute scrotal pain who presented to UHND during the study period were transferred out. Most children (55%) were within the pre-pubertal age group of 5–12 years, which has less likelihood of having TT. This transfer could therefore have been avoided with the improved diagnostic accuracy of the history and clinical examination being augmented by a TWIST score and a DUS. The real clinical impact, however, lies in the orchidectomy rate of children with true TT. At GNCH and SRH the current orchidectomy rates are 19% and 16%, respectively. In this cohort of transferred patients, despite the mean time in UHND being <2h, the final orchidectomy rate was 67%. Peeraully *et al* reported the same effect, where after ‘decentralisation’ of TT management in the Nottingham area, the orchidectomy rate was reduced from 58% to 16%.^26^ Even if a torted testis is ‘saved’, Hamarat *et al* have shown in their reciever operating characteristic analysis, that the risk of testicular volume loss can be predicted with 87.5% sensitivity and 83.9% specificity when the time from the onset of pain to surgery exceeds 5.5h (area under curve = 0.904).^29^ This means that the chance of true salvage of a torted testis transferred to another centre is likely to be very low on long-term follow-up.

The concept of ODNs in the UK was created to increase local capabilities by support and training. This would allow ‘local patients to be treated locally’. If ODNs can fulfil their aims, they will surely improve TT outcomes. It has been shown in multiple reports that the main risk factor for testicular loss is time from the onset of symptoms to treatment. Within this period, it is often the time from the onset of symptoms to arrival at the treating hospital that is the main source of delay. Improved education of the public regarding TT and encouraging early evaluation of testicular pain, making sure that healthcare staff remember to examine the testes in any male with abdominal pain, and improved provision for local surgical intervention without needing to transfer to another hospital will have the most effect in reducing orchidectomy rates.^30^ Campaigns like ‘Save the Ball’ aim to improve public awareness.^31^ A recent meta-analysis reported that for patients presenting within 24h of symptom onset, being transferred to another facility for management led to them being more likely to undergo orchiectomy compared with those treated at their presenting institution (risk ratio 0.35, 95% confidence interval 0.24–0.51; level of heterogeneity (*I*^2^) = 4%).^32^ Once in the treating hospital, it has been shown that protocols to streamline the path from the ED to operating room do not significantly influence orchidectomy rates.^33^ This reiterates that it is the delay in presentation to the treating hospital, and not intrahospital delays, that have the most influence on the risk of orchidectomy. The GIRFT report stresses the importance of revalidation and maintaining skills with the aid of annual continued professional development for regional surgeons. This will allow for local surgical support, if required, for patients presenting to the ED of district general hospitals, and thereby decreasing the need to transfer them elsewhere.

## Conclusions

Improvement in the salvageability of a torted testicle will only happen with better public awareness, encouraging earlier presentation to a healthcare facility, and better local provision for surgical and anaesthetic care that will prevent an onward transfer of these patients. Both at the district general and tertiary hospital levels, in patients that do not have a high clinical risk of TT, evaluation should be aided by clinical risk scores and DUS to reduce the number of unnecessary explorations.

## References

[1] Anderson JB, Williamson RC. Testicular torsion in Bristol: a 25-year review. *Br J Surg* 1988; **75**: 988Y992. 3219547 10.1002/bjs.1800751015

[2] Friedman AA, Ahmed H, Gitlin JS, Palmer LS. Standardized education and parental awareness are lacking for testicular torsion. *J Pediatr Urol* 2016; **12**: 166.e1–8. 10.1016/j.jpurol.2016.01.008 26994588

[3] *GIRFT (Getting it Right First Time) Children and young people: testicular torsion pathway* [Internet]. 2024. https://gettingitrightfirsttime.co.uk/wp-content/uploads/2024/02/Paediatric-testicular-torsion-pathway-guide-FINAL-V1-February-2024.pdf (cited February 2024).

[4] National Confidential Enquiry into Patient Outcome and Death (NCEPOD). *Testicular torsion* [Internet]. 2024. https://www.ncepod.org.uk/2024testiculartorsion.html (cited February 2024).

[5] North East and North Cumbria PCC+SIC Operational Delivery Network [Internet]. 2024. https://www.nenc-pcc-sic-odn.org.uk/about-us/our-aim-strategy-and-vision

[6] Mansbach JM, Forbes P, Peters C. Testicular torsion and risk factors for orchiectomy. *Arch Pediatr Adolesc Med* 2005; **159**: 1167e71. 16330742 10.1001/archpedi.159.12.1167

[7] Zhao LC, Lautz TB, Meeks JJ *et al.* Pediatric testicular torsion epidemiology using a national database: incidence, risk of orchiectomy and possible measures toward improving the quality of care. *J Urol* 2011; **186**: 2009–2013. 21944120 10.1016/j.juro.2011.07.024

[8] Soccorso G, Ninan GK, Rajimwale A, Nour S*.* Acute scrotum: is scrotal exploration the best management? *Eur J Pediatr Surg* 2010; **20**: 312–315. 20577950 10.1055/s-0030-1254150

[9] Murphy FL, Fletcher L, Pease P. Early scrotal exploration in all cases is the investigation and intervention of choice in the acute paediatric scrotum. *Ped Surgery Int* 2006; **22**: 413–416. 10.1007/s00383-006-1681-0 16602024

[10] Gopal M, O’Connor E, McDonald L *et al.* Emergency scrotal exploration in children: is it time for a change in mindset in the UK? *J Pediatr Urol* 2021; **17**: 190.e1–190.e7. 10.1016/j.jpurol.2020.11.029 33317943

[11] Barbosa JA, Tiseo BC, Barayan GA *et al.* Development and initial validation of a scoring system to diagnose testicular torsion in children. *J Urol* 2013; **189**: 1859. 23103800 10.1016/j.juro.2012.10.056

[12] Sheth KR, Keays M, Grimsby GM *et al.* Diagnosing testicular torsion before urological consultation and imaging: validation of the TWIST score. *J Urol* 2016; **195**: 1870–1876. 26835833 10.1016/j.juro.2016.01.101

[13] Qin KR, Qu LG. Diagnosing with a TWIST: systematic review and meta-analysis of a testicular torsion risk score. *J Urol* 2022; **208**: 62–70. 35238603 10.1097/JU.0000000000002496

[14] Kalfa N, Veyrac C, Lopez M *et al.* Multicenter assessment of ultrasound of the spermatic cord in children with acute scrotum. *J Urol* 2007; **177**: 297–301. 17162068 10.1016/j.juro.2006.08.128

[15] McDowall J, Adam A, Gerber L *et al.* The ultrasonographic “whirlpool sign” in testicular torsion: valuable tool or waste of valuable time? A systematic review and meta-analysis. *Emerg Radiol* 2018; **25**: 281–292. 29335899 10.1007/s10140-018-1579-x

[16] *2016 Commissioning guide: Management of paediatric torsion* [Internet]. 2016. https://www.england.nhs.uk/mids-east/wp-content/uploads/sites/7/2018/03/torsion-commissioning-guide.pdf [Updated 2019 Oct, Published 2016 Oct].

[17] Riccabona M, Darge K, Lobo ML *et al.* ESPR uroradiology taskforce–imaging recommendations in paediatric uroradiology, part VIII: retrograde urethrography, imaging disorder of sexual development and imaging childhood testicular torsion. *Pediatr Radiol* 2015; **45**: 2023–2028. 26626757 10.1007/s00247-015-3452-3 PMC4666898

[18] Gopal M, Thattaruparambil V, McLaran P *et al.* Emergency scrotal exploration in children: following a change in mindset in the UK. *J Pediatr Urol* 2023; **19**: 474–476. 37080795 10.1016/j.jpurol.2023.03.039

[19] Friedman N, Pancer Z, Savic R *et al.* Accuracy of point-of-care ultrasound by pediatric emergency physicians for testicular torsion. *J Pediatr Urol* 2019; **15**: 608.e1–608.e6. 10.1016/j.jpurol.2019.07.003 31455581

[20] Mori T, Ihara T, Nomura O. Diagnostic accuracy of point-of-care ultrasound for paediatric testicular torsion: a systematic review and meta-analysis. *Emerg Med J* 2023; **40**: 140–146. 35523539 10.1136/emermed-2021-212281

[21] Sharma A, Nathan A, Rossiter M *et al.* Scrotal point-of-care ultrasonography: a UK cross-speciality pilot training course evaluation. *BJU Int* 2023; **132**: 645–648. 37539771 10.1111/bju.16146

[22] Cornford P, Foster L, Hodgson D *et al*. *Urology curriculum. The Intercollegiate Surgical Curriculum Programme (ISCP)* [Internet]. 2021. https://www.iscp.ac.uk/media/1112/urology-curriculum-aug-2021-approved-oct-20.pdf (cited August 2021).

[23] The Intercollegiate Surgical Curriculum Programme (ISCP). *General surgery curriculum* [Internet]. 2021. Available from https://www.iscp.ac.uk/media/1103/general-surgery-curriculum-aug-2021-approved-oct-20v3.pdf (cited August 2021).

[24] Lewis S, Hopkins L, Evans T *et al.* Testicular torsion treatment: the horns of a dilemma? *Ann R Coll Surg Engl* 2020; **102**: 49–53. 10.1308/rcsann.2019.0150 PMC6937606 31755741

[25] Getting it Right First Time (GIRFT). *GIRFT programme national speciality report by Professor Simone E Kenny* [Internet]. 2021. https://gettingitrightfirsttime.co.uk/wp-content/uploads/2022/09/PaediatricSurgeryReport-Sept21w.pdf (cited February 2021).

[26] Peeraully R, John M, Ellis R, Green S *et al.* Does decentralisation of surgical management improve outcomes for paediatric testicular torsion? *J Pediatr Urol* 2022; **18**: 302.e1–302. 10.1016/j.jpurol.2022.03.020 35410806

[27] Royal College of Anaesthetists. *Guidelines for the provision of paediatric anaesthesia services 2023* [Internet]. 2023. https://www.rcoa.ac.uk/gpas/chapter-10#chapters-1.1 (cited January 2023).

[28] Royal College of Surgeons in England. *Standards for non-specialist emergency surgical care of children* [Internet]. 2015. https://www.rcseng.ac.uk/library-and-publications/rcs-publications/docs/standards-for-non-specialist-emergency-surgical-care-of-children/ (cited November 2015).

[29] Hamarat MB, Dönmez Mİ, Sezgin T *et al.* Testicular volume loss in the long-term follow-up after surgical detorsion of the testis. *Pediatr Surg Int* 2022; **38**: 907–911. 35366086 10.1007/s00383-022-05118-x

[30] Ryan KA, Folkard SS, Bastianpillai C, Green JSA. The management of testicular torsion in the UK: how can we do better? A national quantitative and qualitative analysis of the factors affecting successful testicular salvage. *J Pediatr Urol* 2020; **16**: 815.e1. 10.1016/j.jpurol.2020.08.018 32933873

[31] The Urology Foundation. *Testicular torsion* [Internet]. 2024. https://www.theurologyfoundation.org/news/624-save-the-ball (cited January 2024).

[32] Kwenda EP, Locke RA, DeMarco RT, Bayne CE. Impact of hospital transfer on testicular torsion outcomes: A systematic review and meta-analysis. *J Pediatr Urol* 2021; **17**: 293. 10.1016/j.jpurol.2021.01.038 33610457

[33] Arevalo MK, Sheth KR, Menon VS *et al.* Straight to the operating room: an emergent surgery track for acute testicular torsion transfers. *J Pediatr* 2018; **192**: 178–183. 29246339 10.1016/j.jpeds.2017.09.009 PMC5737783

